# Does the edge effect impact on the measure of spatial accessibility to healthcare providers?

**DOI:** 10.1186/s12942-017-0119-3

**Published:** 2017-12-11

**Authors:** Fei Gao, Wahida Kihal, Nolwenn Le Meur, Marc Souris, Séverine Deguen

**Affiliations:** 1 EHESP Rennes, Sorbonne Paris Cité, Paris, France; 2 LIVE UMR 7362 CNRS (Laboratoire Image Ville Environnement), University of Strasbourg, 6700 Strasbourg, France; 3 L’équipe REPERES, Recherche en Pharmaco-épidémiologie et recours aux soins, UPRES EA-7449, Rennes, France; 4 IRD, UMR_D 190 “Emergence des Pathologies Virales” (IRD French Institute of Research for Development, Aix-Marseille University, EHESP French School of Public Health), Marseille, France; 5 Department of Quantitative Methods for Public Health, EHESP School of Public Health, Avenue du Professeur Léon Bernard, 35043 Rennes, France; 6 Department of Social Epidemiology, Sorbonne Universités, UPMC Univ Paris 06, INSERM, Institut Pierre Louis d’Epidémiologie et de Santé Publique (UMRS 1136), Paris, France

**Keywords:** Edge effect, Potential spatial accessibility of healthcare professionals, E2SFCA algorithm, Geographic information systems, Spatial analyses, Pregnant women

## Abstract

**Background:**

Spatial accessibility indices are increasingly applied when investigating inequalities in health. Although most studies are making mentions of potential errors caused by the edge effect, many acknowledge having neglected to consider this concern by establishing spatial analyses within a finite region, settling for hypothesizing that accessibility to facilities will be under-reported. Our study seeks to assess the effect of edge on the accuracy of defining healthcare provider access by comparing healthcare provider accessibility accounting or not for the edge effect, in a real-world application.

**Methods:**

This study was carried out in the department of Nord, France. The statistical unit we use is the French census block known as ‘IRIS’ (Ilot Regroupé pour l’Information Statistique), defined by the National Institute of Statistics and Economic Studies. The geographical accessibility indicator used is the “Index of Spatial Accessibility” (ISA), based on the E2SFCA algorithm. We calculated ISA for the pregnant women population by selecting three types of healthcare providers: general practitioners, gynecologists and midwives. We compared ISA variation when accounting or not edge effect in urban and rural zones. The GIS method was then employed to determine global and local autocorrelation. Lastly, we compared the relationship between socioeconomic distress index and ISA, when accounting or not for the edge effect, to fully evaluate its impact.

**Results:**

The results revealed that on average ISA when offer and demand beyond the boundary were included is slightly below ISA when not accounting for the edge effect, and we found that the IRIS value was more likely to deteriorate than improve. Moreover, edge effect impact can vary widely by health provider type. There is greater variability within the rural IRIS group than within the urban IRIS group. We found a positive correlation between socioeconomic distress variables and composite ISA. Spatial analysis results (such as Moran’s spatial autocorrelation index and local indicators of spatial autocorrelation) are not really impacted.

**Conclusion:**

Our research has revealed minor accessibility variation when edge effect has been considered in a French context. No general statement can be set up because intensity of impact varies according to healthcare provider type, territorial organization and methodology used to measure the accessibility to healthcare. Additional researches are required in order to distinguish what findings are specific to a territory and others common to different countries. It constitute a promising direction to determine more precisely healthcare shortage areas and then to fight against social health inequalities.

## Background

Equitable distribution of health resources is a key priority for health professionals and policy makers worldwide; reducing health inequalities has long been of concern to community and public health planners [1–4]. Access to healthcare, as one potential driver of health inequalities, is at the heart of public health policy and is internationally recognized as a key goal in meeting the essential health needs of individuals [5–8].

Access to healthcare varies across space due to the uneven distribution of both healthcare providers and consumers, and the impact of geographical location on health is increasingly being examined. Various studies in Europe (including France) have shown unequal distribution of health service resources [9]. With heightened interest in residential neighborhood the characteristics that could influence health behaviors and outcomes, spatial accessibility and availability indices are being used in epidemiological studies more and more [10–15]. As a measure for determining those areas having inadequate levels of health service provision, spatial accessibility of health services refers to relative access to health services in a given location, which is influenced primarily by travel distance (or travel time) and the spatial distribution of health service providers and consumers [16–18]. Most studies examining the geographical accessibility of healthcare and health-related services have suggested a growing range of indices, including Physician Population Ratio, nearest distance, shortest time, cumulative opportunity and the gravity model [5, 19–26]. Recent methodological developments in this field have emerged in international research, including Enhanced 2-Step Floating Catchment Area method (E2SFCA) [27], which provides a summary measure of two important and related components of access: volume of services provided relative to population size, and proximity of services provided relative to population location.

In addition, one methodological limitation often mentioned in research considering accessibility concerns the fact that studies failed to include behavior outside the study area [17, 28–39]. Known as the edge effect, it is central to this paper. Edge effect occurs “when the study area is defined by a border which does not actually prevent travel across the border” [40] and people are free to travel beyond that border to receive healthcare goods and services. Arbitrary administrative boundaries (such as census tracts or block groups) are often used without consideration that resources beyond a given boundary are likely to affect behaviors within a given spatial unit [35]. This means that any geographic distribution or spatial interaction occurring within the spatial unit may extend beyond its boundaries [30]. More precisely, edge effects manifest when the boundaries of the study area affect a given spatial measurement and lead to the distortion of estimates [35, 41]. Interestingly, although most studies do mention potential errors caused by the edge effect, many acknowledge their mistake in neglecting to consider this in the spatial analyses they have undertaken within a finite region [42]. Because this can result in areas close to the boundary being classified as having poor geographic access even though they may in fact be proximate to resources across the boundary, many research projects have hypothesized that failure to accounting for edge effect will lead to considerable biases [34–37], even under-reporting [17, 28, 29, 31, 43] of accessibility to facilities.

Although edge effect is a well-documented phenomenon, researches choosing this issue as the main subject used for most of the time distance/travel time measure [34, 35], or availability measures such cumulative index [28, 34, 35, 38, 43]. Focusing on E2SFCA method, the edge effect is frequently observed in studies measuring the spatial accessibility to healthcare providers. More and more studies have corrected for edge effects [32, 33]. However, to the best of our knowledge, very few studies based on E2SFCA have focused on edge effect in a real-world application with a view to quantifying its effect on the accuracy of defining health service access.

In this context, our study compares health service accessibility when accounting or not for the edge effect, taking into account that patients may overcome geographical boundaries, choosing to consult health professionals in neighboring departments. The geographical accessibility indicator used to quantify spatial accessibility is the *Index of spatial accessibility* (*ISA*), based on the E2SFCA algorithm. ISA was previously developed by our team for the pregnant women population, focusing on the three types of healthcare professionals (GP, midwife and gynecologist) involved during the pregnancy [44]. Conducted in the department of Nord at French census block spatial scale, our study aimed to quantify edge effect bias using the ISA index, and investigate the impact on spatial analysis results.

Besides, it is well documented that levels of accessibility and utilization of healthcare are related with socio-economic distress level and geographical factors [45–48]. Consequently, in our study, we investigated the urban–rural disparity of ISA as well as the relationship of ISA with socioeconomic distress variables, both when offer and demand beyond the area of study are excluded or included. The underlying questions are: Would the association between socioeconomic factor and accessibility be biased by ignoring spatial interaction occurring between the spatial unit and its neighborhood? Would the difference of accessibly between urban/rural areas be accentuated?

## Methods

### Data and measures

#### Study setting and statistical unit

This study was carried out in the department of Nord, located in the north of France, close to the Belgian border. Analysis was conducted at French census block level (known as IRIS: “Ilots Regroupés pour l’Information Statistique”) defined by the National Institute of Statistics and Economic Studies (INSEE) [49], which is the smallest infra-urban level for which census data is available. There are 1346 IRIS in the department of Nord.

#### Neighborhood characteristics

Two types of neighborhood characteristic were used at census-block level:

(i) *Degree of urbanization (rural/urban)* Each IRIS was classified as urban or rural according to the classification established by the national census bureau. These data are openly available from (https://www.insee.fr/fr/information/2017499) [49].
(ii) *Level of socioeconomic distress* According to previous work on social health inequality [50], we selected five variables from the 2006 French census (https://www.insee.fr/fr/information/2017499) [49] to characterize the neighborhood socioeconomic level: low level of educational attainment, women’s unemployment rate, single parent families, non-homeowner, and insecure employment situation (see variables definition in “Appendix I”).

#### Health professionals

The postal addresses of GPs, midwives and gynecologists were obtained from the French state health insurance website (http://www.ameli-sante.fr) in 2014 [51]. To assess the edge effect, we considered the health professional offer both within and outside of the department of Nord. Service providers were represented by their geocoded professional addresses (latitude, longitude), obtained through Batch Geocoder (http://dehaese.free.fr/Gmaps/testGeocoder.htm). Eight general practitioners and one obstetrical gynecologist were excluded from the analysis due to low quality of professional postal addresses. No georeferencing quality difference was detected between adjacent department and Nord Department. Further methodological details are available elsewhere [44].

#### Index of spatial accessibility (ISA)

ISA is an indicator which measures healthcare service accessibility.

The ISA is based on the E2SFCA method, a method which maintains the advantages of a gravity model while being easier to interpret, since it represents a derived form of a Physician Population Ratio. As the name suggests, two steps must be performed:

- **Step 1** For each provider in location *k*, look up all population locations of IRIS *i* within a catchment, and within a predefined distance *d*_*ik*_ from location k. A distance decay function is applied within a catchment. *w(d*_*ik*_ *)* is the weight quantifying travel time between IRIS *i* and healthcare provider *k.* Sum up all population sizes (Pi) within that catchment area to compute the provider-to-population ratio (*R*_*k*_):
  1 $$R_{k} = \frac{1}{{\mathop \sum \nolimits_{{d_{jk} < d_{max} }} P_{i} *w\left( {d_{ik} } \right)}}$$
- **Step 2** For each population location *i*, look up all provider locations *k* that are within the catchment from location *i*. Sum up all *R*_*k*_ for the catchment area to calculate the Index of spatial accessibility (*ISA*_*i*_) at location *i*:
  2 $$ISA_{i} = \mathop \sum \limits_{{{\text{d}}_{\text{ij}} \le {\text{d}}_{ \text{max} } }} w\left( {d_{ij} } \right)R_{k}$$
  ISA takes into account:

(i) The latitude and longitude of each healthcare professional.
(ii) The centroids of residential buildings for each IRIS (Residential buildings came from BD TOPO^®^ and was provided by the *Institut National de l’Information Géographique et Forestière (French National Geographic Institute)* [52]). And
(iii) Car travel time, calculated by Google Maps. We used the FILENAME statement and the URL access method within SAS to access Google Maps, and extracted both the driving time and distance each time the site was accessed [44, 53].

We estimated an ISA for GPs, gynecologists and midwives, separately. A composite ISA relying on principal component analysis was also calculated, describing overall accessibility of the three types of healthcare professionals. Further details of the method developed for ISA estimation are given in [44].

#### Decay function and travel time threshold

We defined the time threshold according to figures already published by *the French Institute for research and information in health economics* for general practitioners [54]:

- less than 5 min’ travel: fully access to healthcare providers (*w* = 1)
- more than 15 min’ travel: no access to healthcare providers (*w* = 0).
- between 5 and 15 min: partial access to healthcare providers (*w* is defined by a continuous decay function [Eq. (3)] with the weighting factor equal to 1.5 [55])

3 $$w = \frac{{\left( {15 - d} \right)}}{{\left( {15 - 5} \right)}}e^{1.5}$$

We based the threshold of the two other healthcare professionals on general practitioners’ results: the nearest travel time to general practitioner is lower than 5 min and between 5 and 15 min for 88 and 12% of the population, respectively; we used these proportions to define the threshold for two other health professionals: 15 and 34 min for gynecologists and 17 and 34 min for midwives.

**Fig. 1:**
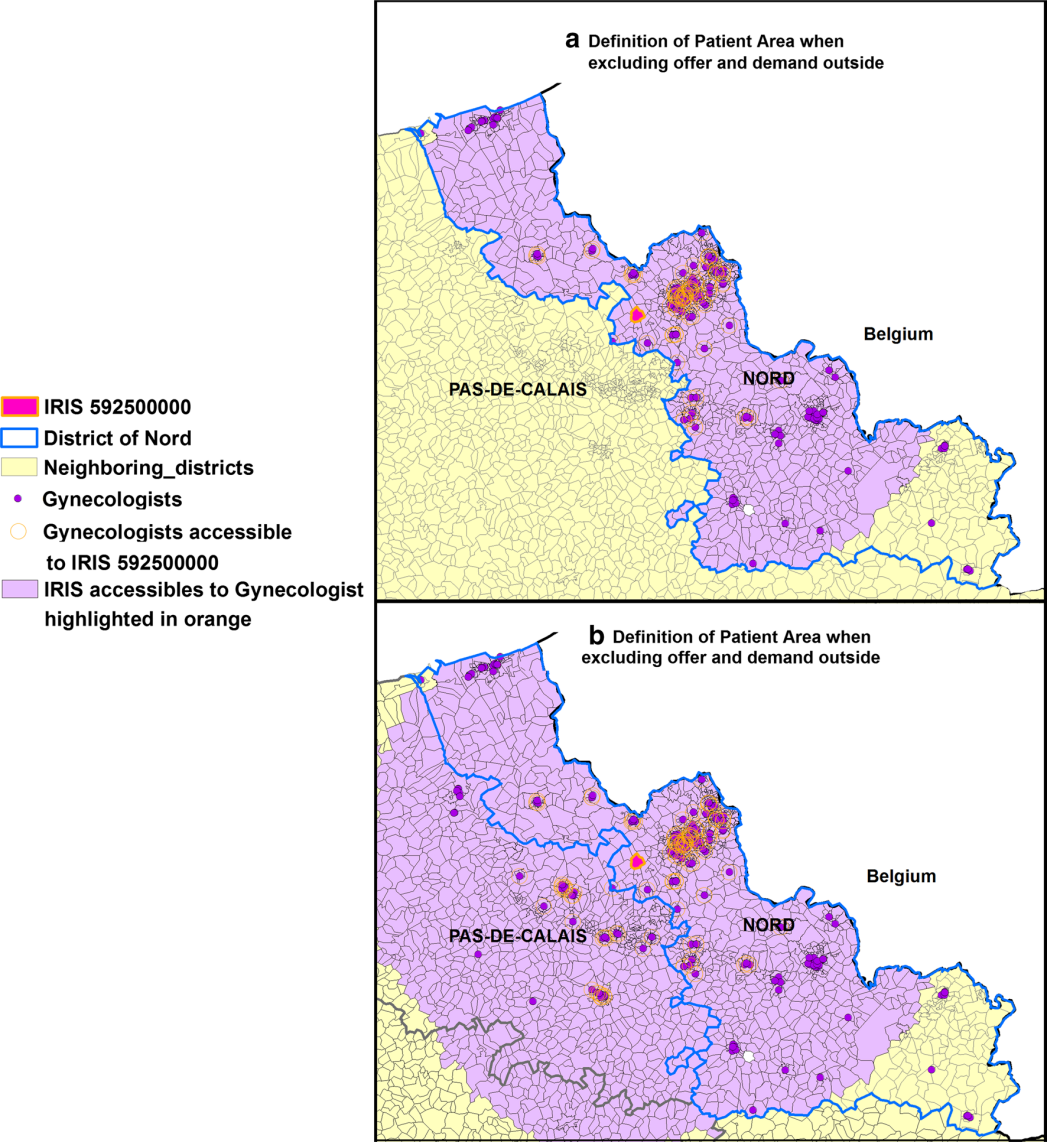
Definition of “patient area” when including and excluding offer and demand outside. Focus on the IRIS named “Fournes-en-Weppes”- (IRIS no. 592 500 000), the Nord department are circled in blue, whereas neighboring IRIS from the three departments of Somme, Aisne and Pas-de-Calais are yellow.** a**) without consideration of offer and demand beyond the boundary;** b**) with consideration of offer and demand beyond the boundary

 Figure 1 provides an illustration of the impact of including offers and demands outside the study area defining what we call the “patient area” or catchment. This illustration deals with gynecologists only for the IRIS named “Fournes-en-Weppes” (IRIS no. 592 500 000), with keys for reading.

Keys for reading Fig. 1 and fully understanding the principle of edge effect:

- Figure 1a—study area without consideration of offer and demand beyond the boundary
- All 218 gynecologists are represented by dark purple dots. The IRIS “Fournes-en-Weppes” is highlighted in fuchsia and circled in orange. We count 146 gynecologists accessible by car within 34 min of Fournes-en-Weppes, within the study area. The 1201 IRIS are highlighted in purple forms the “patient area” of the 146 gynecologists (circled in orange). Figure 1b—study area with consideration of offer and demand beyond the boundary

With edge effect, the residents of Fournes-en-Weppes could reach 181 gynecologists (an additional 35 from outside) within 34 min by car. However, they must share these with 2203 IRIS (1001 IRIS from outside). “Patient area” IRIS are colored purple.

### GIS methods

We began by quantifying a global ISA spatial autocorrelation, separately with, and without, consideration of offer and demand beyond the department of Nord, based on Moran’s I statistic (calculated by means of the distance matrix) [56–58]. Spatial autocorrelation can be defined as the coincidence of value similarity and locational similarity [59]. Positive spatial autocorrelation therefore exists where the high or low values of a random variable tend to be spatially clustered, with negative spatial autocorrelation existing where geographical areas tend to be surrounded by neighbors having highly dissimilar values. The values of the Moran’s I statistic range from − 1 to + 1.

Next, a Local Indicator of Spatial Autocorrelation (LISA) was applied. More precisely, Moran’s diagram was produced in order to reveal the types of spatial relationship between a geographic unit and its neighboring area.

Four types of LISA can be detected: High–High (HH): high level of ISA in both a given IRIS and in its neighbors and Low–Low (LL): low level of ISA in both a given IRIS and in its neighbors, characterizing a positive association; High–Low (HL): high level of ISA in a given IRIS, whereas its neighbors have a low level of ISA and Low–High (LH): low level of ISA in a given IRIS, whereas its neighbors have high level of ISA, characterizing a negative association.

### Statistical analysis

#### Classification

In order to analyze ISA variations when offer and demand outside are included, the 1346 IRIS making up the Nord department are divided into three classes, named *improved, unchanged* and *deteriorated*. These classes were constructed according to the results obtained using the simple linear regression model, where Y and X correspond to the ISA estimated with and without taking into account offer and demand across the boundary, respectively (see “Appendix II”).

#### Statistical associations

ISA’s composite values when offer and demand beyond the boundary were then cross-referenced with the individual variables of socioeconomic distress mentioned in the data section. The statistical significance of the relation was tested using a simple linear regression where Y and X were the ISA index and one of the socioeconomic variables, respectively. The α-risk was set at 5%.

#### Strategy and the statistical analysis plan

Preliminary work was carried out to study ISA variation when offer and demand outside are excluded or included, and the spatial distribution of this variation. To quantify overall and local autocorrelation of ISA in the two cases, the GIS method was then applied. Following this, we analyzed the ISA variation for urban and rural zones, separately. Finally, we compared the relationship between the socioeconomic distress variable and ISA, to find out whether there is an impact when studying the association, both when excluding and including healthcare offer and demand outside the area of study, to account for a deficiency in analysis termed the “edge effect”.

## Results

### Descriptive results

When excluding healthcare providers outside the department boundary, we geolocalized 2590 GPs, 143 midwives and 218 gynecologists. In order to include offer and demand beyond outside, we added 493 GPs, 60 midwives and 78 gynecologists from the neighboring area who were capable of providing services to those residing in the department of Nord. Ignoring the offer beyond the department led to an 18% decrease in the total number of health professionals potentially available; this decrease reaches 30% when focusing on midwives (Table 1).

**Table 1:** Number of health professionals by medical specialty

Medical speciality of health professional	Number of healthcare providers			Number of IRIS of “patient area”			
	Department of Nord	Neighboring IRIS	% increase	Department of Nord	Average population	Neighboring IRIS	Average population
GPs	2590	493	16	1346	1905	1362	1076
Midwives	143	60	30	1346	386	2425	187
Gynecologists	218	78	26	1346	986	2583	484
Total	2951	631	18	–	–	–	–

After calculation of travel time via Google Maps, when including offer and demand beyond the boundary, “patient area” is not restricted to the 1346 IRIS of the department of Nord. In all, 1362, 2425 and 2583 IRIS in the departments of Pas-de-Calais, Oise, Somme, Aisne and Ardennes are added to the ISA calculation for GPs, midwives and gynecologists respectively (Table 1). The “average population” columns show that les IRIS neighboring have lower population density than IRIS Nord.

The descriptive statistics of the ISA when offer and demand beyond the study area are included or excluded are presented in Table 2. Mean and standard deviation are slightly below when offer and demand outside are taken into account, whichever health professionals are included. The two-means comparison is only statistically different for ISA gynecologist (*p* < 0.00).

**Table 2:** Descriptive statistics of ISA when accounting or not for the edge effect—North department

	When not accounting for the edge effects			When accounting edge effects		
	Min^¥^	Mean (SD*)	Max^ɸ^	Min^¥^	Mean (SD*)	Max^ɸ^
GPs	1.67	93.42 (35.74)	245.88	1.67	92.98 (35.17)	245.88
Midwives	0	22.64 (11.57)	50.29	0	21.16 (11.06)	49.90
Gynecologists	0	22.47 (9.51)	43.76	0	20.20 (8.62)	41.46
Composite index**	0.40	39.38 (14.19)	91.43	0.41	39.40 (13.92)	91.98

ISA GPs is expressed per 100,000 inhabitants, ISA Midwives per 100,000 women inhabitants aged between 15 and 44 and ISA gynecologists per 100,000 women inhabitants

*Standard deviation

**Composite ISA resulting from the principal component analysis

^**¥**^Minimum

^**ɸ**^Maximum

### Spatial distribution of ISA at IRIS level

**Fig. 2:**
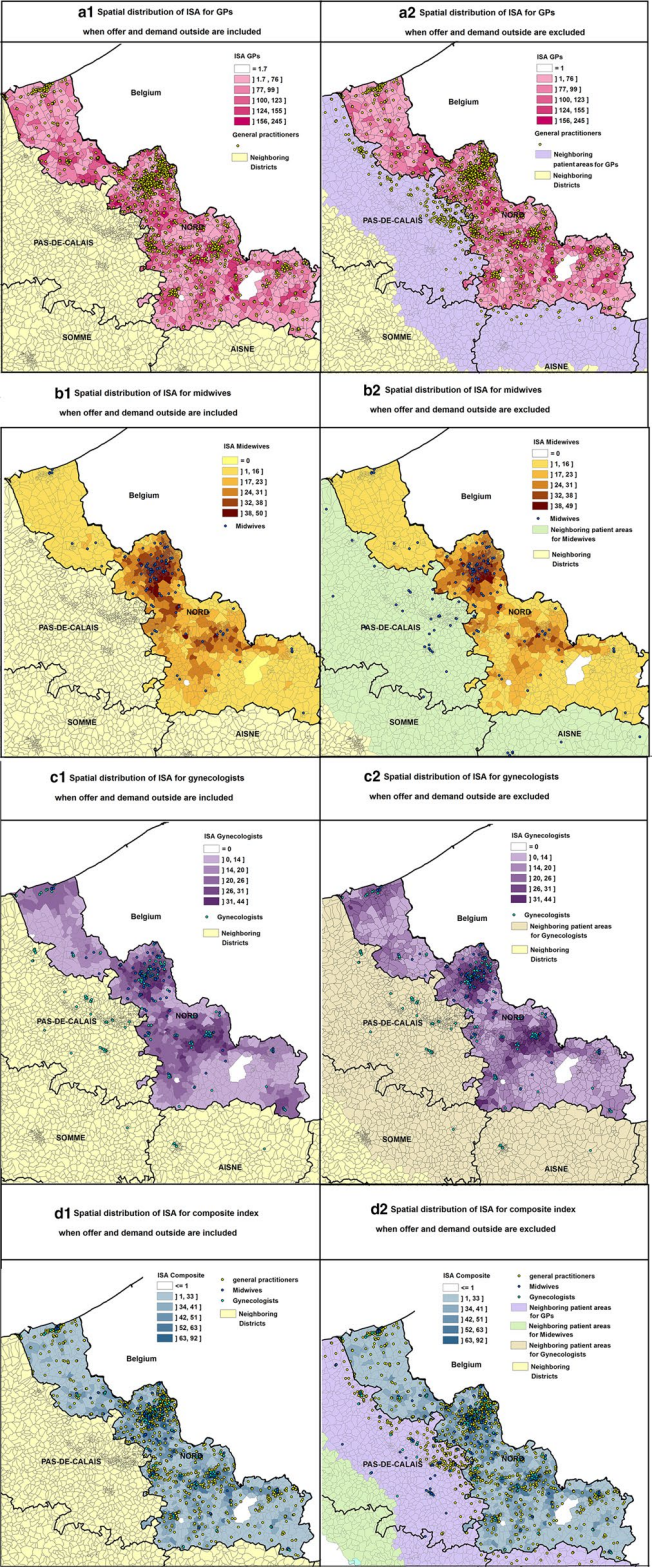
Spatial distribution of ISA when offer and demand outside are included or excluded. ISA distribution is showed for GPs (**a**), midwives (**b**) and gynecologists (**c**) and combined in the composite index (**d**). For each map, neighboring departments are colored in yellow and the department of Nord is colored using a graduated approach (according to Jenks’ Natural Breaks), showing different ISA scales at IRIS level, expressed per 100,000 inhabitants. The 1362, 2425 and 2583 Neighboring IRIS added to the ISA calculation for GPs, midwives and gynecologists when edge effect included are colored purple, green and khaki respectively

Spatial distributions of ISA for GPs (a), midwives (b) and gynecologists (c) considered separately, and combined in the composite index (d) when offer and demand beyond the department of Nord are included or not (Fig. 2). The maps show minor changes: ISA distributions in the two cases are fairly similar. Changes appear mainly in those IRIS located close to boundaries.

### Accounting for edge effect

In order to focus on ISA variation when offer and demand beyond the study area were included, we distributed the 1346 IRIS into three classes: improved, unchanged and deteriorated according to simple linear regression results (presented with more detail in “Appendix II”).

**Fig. 3:**
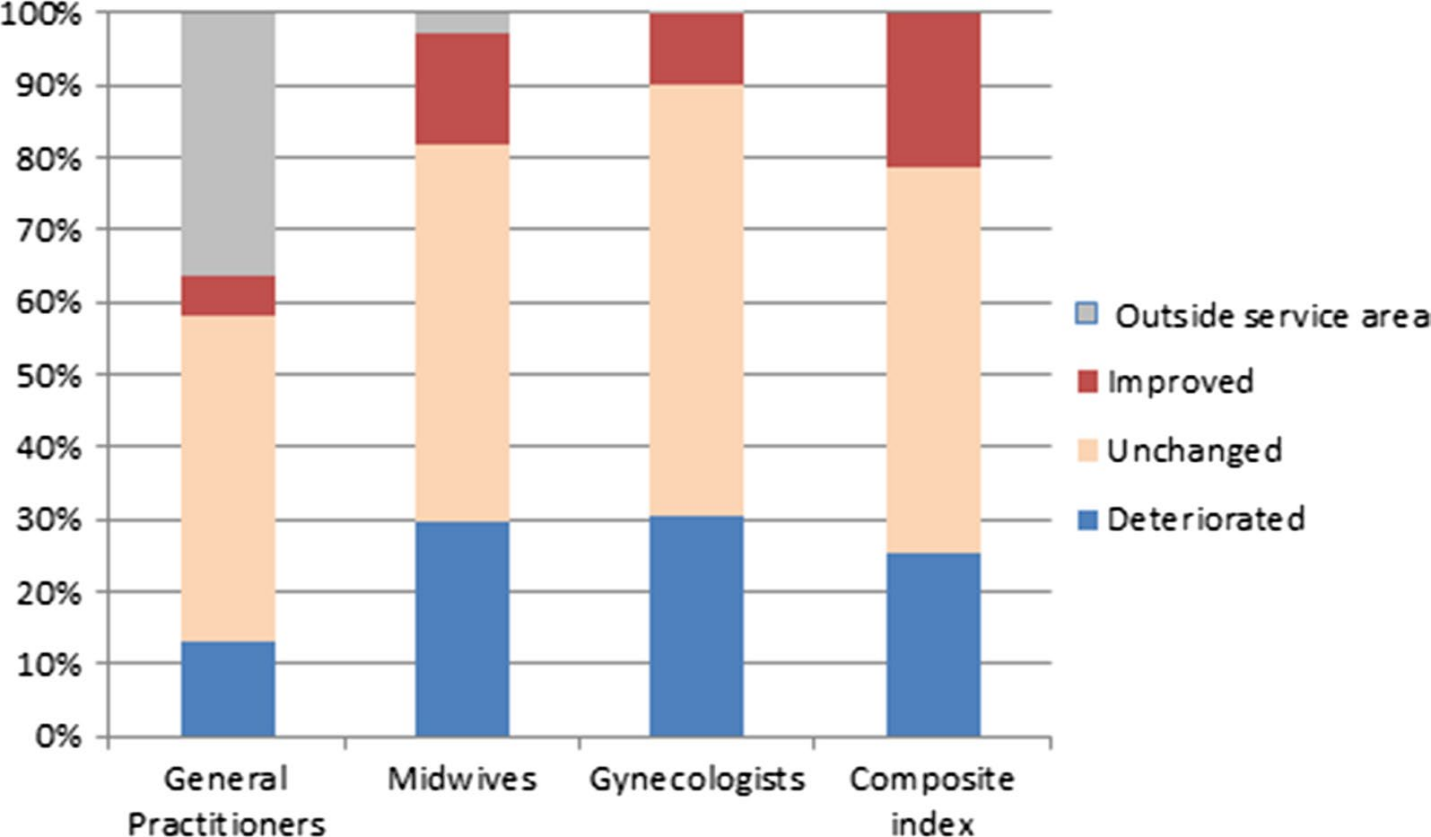
Percentage of residential IRIS having improved/unchanged/deteriorated accessibility when accounting for edge effect

Figure 3 shows that when accounting for healthcare provider source and patient needs outside the area of Nord the percentage of IRIS having decreased ISA is larger than those with increased ISA (13.15 vs. 5.50% for GPs; 29.79 vs. 15.68% for midwives and 30.46 vs. 9.88% for gynecologists). Many past researches have hypothesized that failure to accounting for edge effect will lead to considerable under-reporting of accessibility to facilities. We obtain the exact opposite findings. The composite ISA which give an overall view of accessibility to various types of health professionals is subject to a slight edge effect (25.33% deteriorated and 21.55% improved). Those IRIS too far from boundaries to be affected are colored in grey (“outside service area” in the key).

**Fig. 4:**
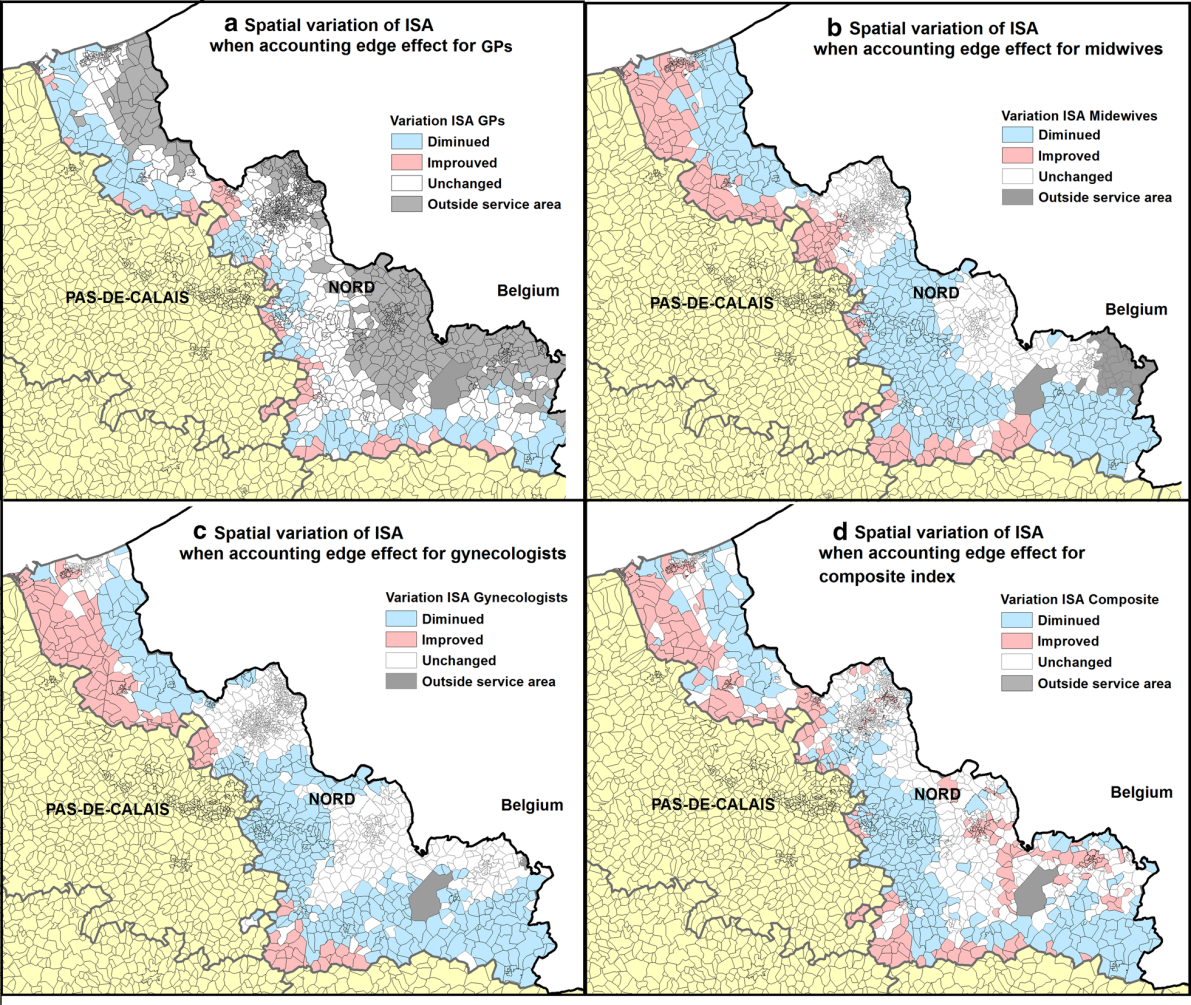
Spatial variation of ISA when including offer and demand beyond the department of Nord. Variation is displayed for GPs (**a**), midwives (**b**) and gynecologists (**c**) considered separately, along with the composite index (**d**). All IRIS that are too far from boundaries (by car travel time) to be affected are shaded grey

It can be observed in Fig. 4 that IRIS where GPs ISA changed are mainly located close to the boundaries. Conversely, only 36 IRIS where midwives ISA are not impacted as a result of distance, and 2 IRIS for gynecologists ISA. The white zone does not mean that they are not subject to edge effect, but rather reveal the existence of a kind of “balance”: people from this zone could reach more healthcare professionals beyond the department of Nord but at the same time they must share health resources with residents from neighboring departments. Their accessibility score therefore remains relatively stable.

When focusing on composite ISA, results reveal that all IRIS are subject to edge effect. Most of the IRIS located close to the border and in the agglomeration area (such as Roubaix, Anzin, Maubeuge and Saint-Pol-sur-Mer) saw their ISA improved. However, more IRIS have a deteriorated ISA (25.3%) than an improved ISA (21.5%).

### Spatial analysis of ISA

The result of Moran’s test for the composite ISA reveal significant spatial autocorrelation (I = 0.73 when offer and demand beyond the study area are included, and I = 0.74 when excluded—*p* = 0.0001, pseudo-significance values based on a permutation approach [56]). This means that the IRIS which have a high level of healthcare accessibility are more often located close to other IRIS having a high ISA score in the two cases than they were if this distribution were random.

**Fig. 5:**
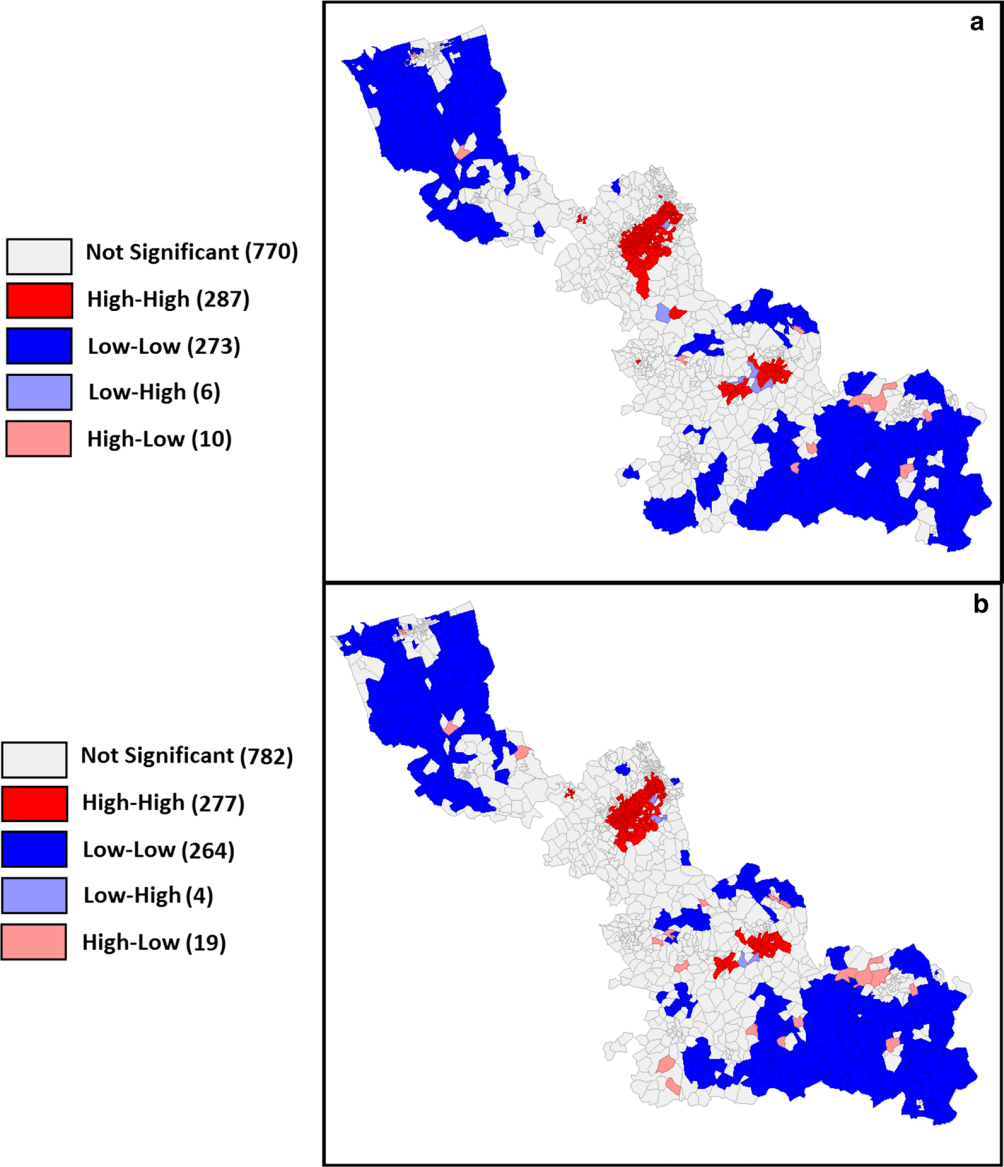
LISA cluster map of composite ISA when accounting or not for the edge effect. When not accounting for the edge effect (**a**) and when accounting for the edge effect (**b**)

Figure 5 shows the mapped results of the LISA statistics calculations. According to the results obtained from the LISA statistics, when excluding the offer and demand beyond the boundary, the 1346 IRIS are distributed as follows (Table 3): 287 HH-type (high level surrounded by high levels), 273 LL-type (low level surrounded by low levels). Despite some minor differences, we found similar distribution of LISA statistics: 277 HH-type, 264 LL-type.

**Table 3:** Descriptive statistics of composite ISA in the IRIS types obtained by LISA statistics

LISA statistic level types	HH	LL	LH	HL	NS	Total
*Number of IRIS when accounting for the edge effect*	*287*	*273*	*6*	*10*	*770*	*1346*
*%*	*21.3%*	*20.3%*	*0.4%*	*0.7%*	*57.2%*	
ISA composite						
Minimum	40.1	0.4	33.8	36.3	7.0	
Mean	57.0	24.3	36.3	45.1	38.1	
Standard deviation	8.7	7.9	2.0	5.2	10.1	
Maximum	91.4	39.8	38.8	58.8	77.8	
*Number of IRIS when not accounting for the edge effect*	*277*	*264*	*4*	*19*	*782*	*1346*
*%*	*20.6%*	*19.6%*	*0.3%*	*1.4%*	*58.1%*	
ISA composite						
Minimum	41.3	0.4	6	39.7	7.2	
Mean	57.2	24.6	33.5	45.2	38.2	
Standard deviation	8.7	7.7	36.3	4.2	9.7	
Maximum	92.0	39.2	2.6	54.4	76.7	

Results when accounting or not for the edge effects are shown separately

### Comparative analysis of urban and rural ISA variation with edge effect

**Fig. 6:**
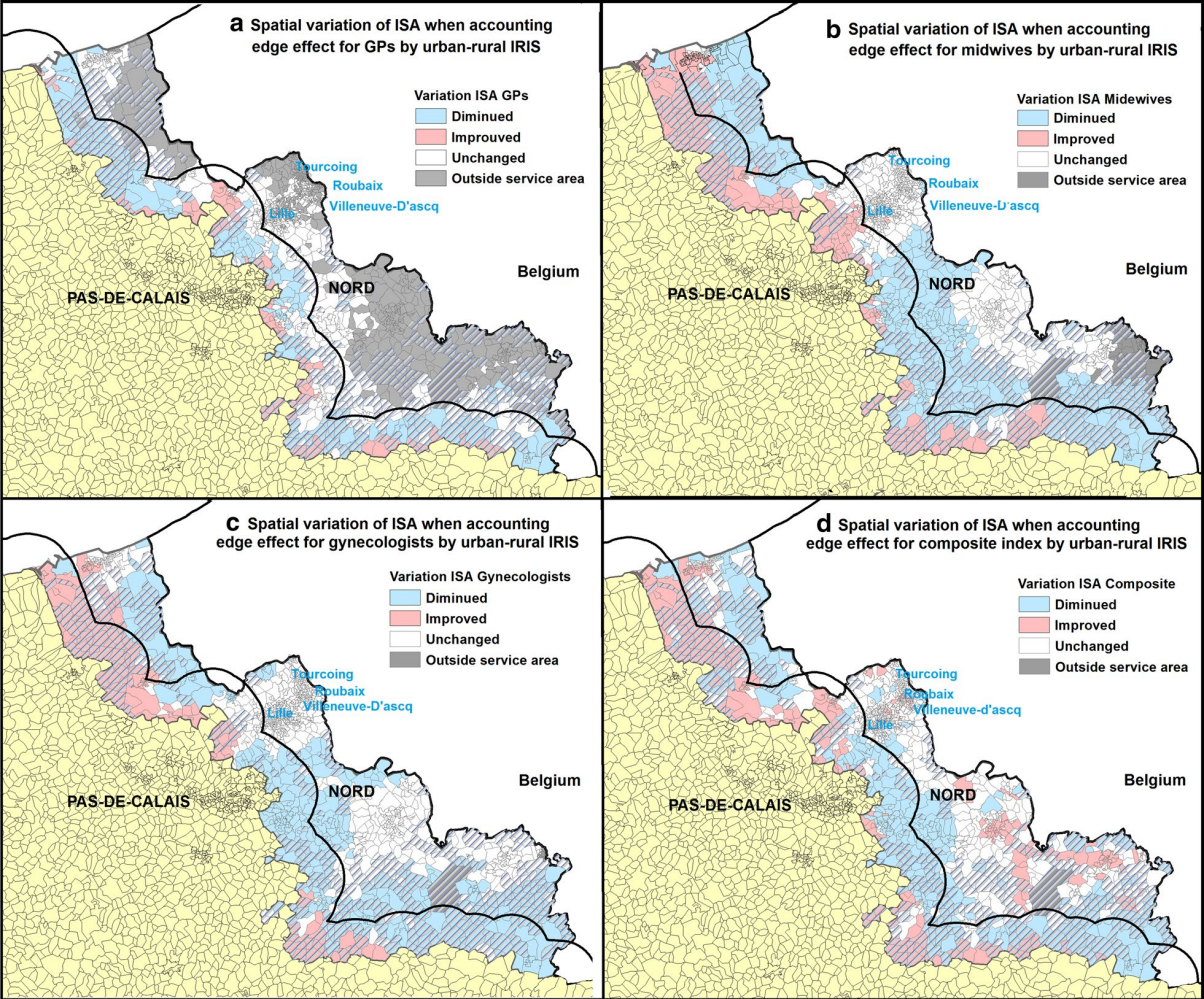
Spatial variation of ISA and the distribution of urban/rural IRIS. ISA variation is showed for GPs (**a**), midwives (**b**) and gynecologists (**c**) and the composite index (**d**). Rural IRIS are hatched. We created a 10KM buffer zone around the boundaries

Figure 6 shows the ISA variation when accounting for the edge effect and the distribution of urban IRIS and rural (hatched) IRIS. Most IRIS in the department of Nord are urban (1030 urban vs. 336 rural), concentrated around several densely-populated areas close to major cities such as Lille, Roubaix, Tourcoing and Villeneuve d’Ascq (Fig. 6). Using a 10 km buffer zone around the boundaries, we estimated that 180 rural IRIS (54% of total rural IRIS) and 304 urban IRIS (just 29% of total urban IRIS) were near the Nord Pas-de-Calais and the Nord Aisne border.

**Fig. 7:**
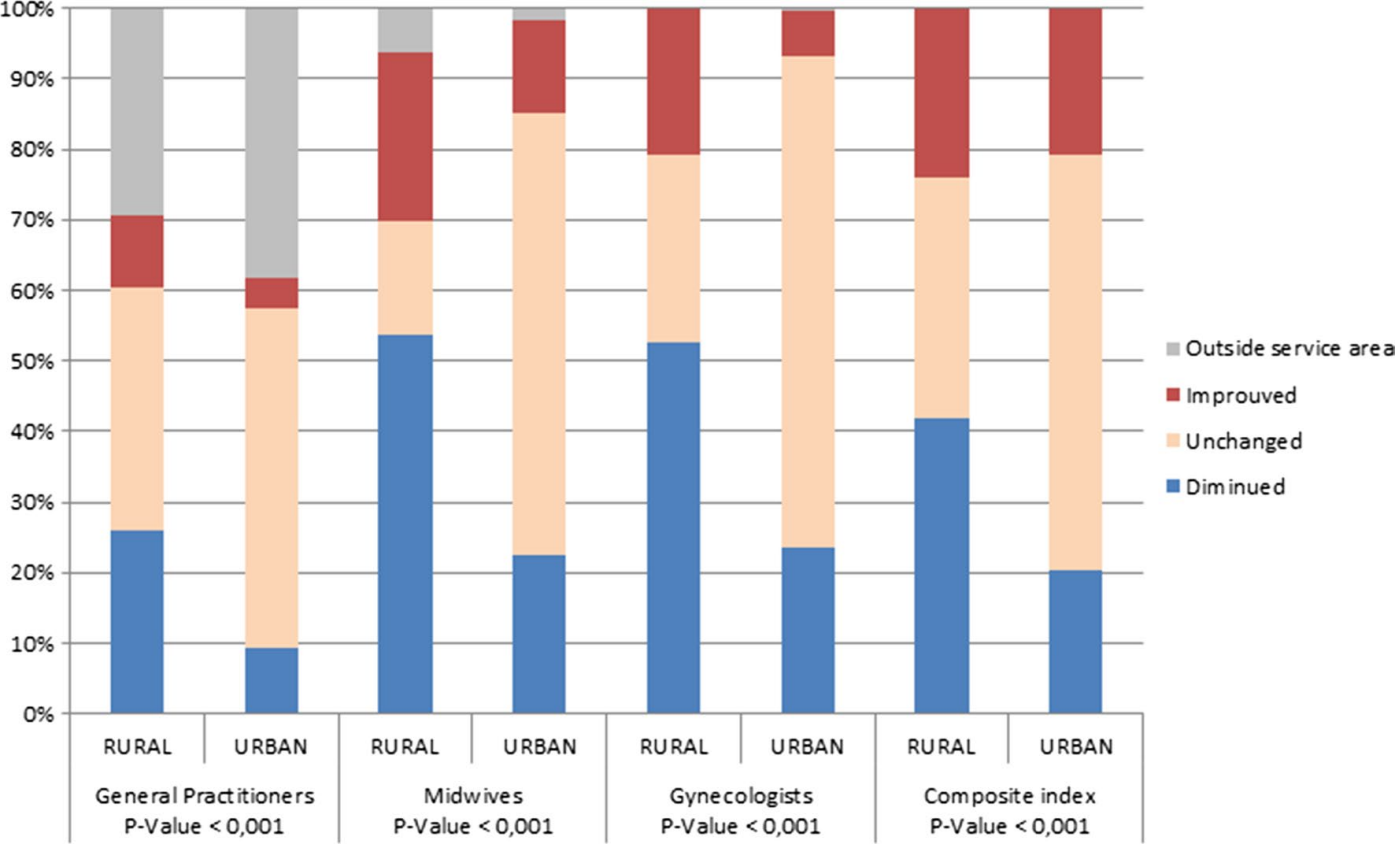
Percentage of Urban/Rural IRIS having improved/unchanged/deteriorated accessibility. ISA variation with edge problem corrected. The *p* value is determined by Chi-square test

Figure 7 shows the percentage of urban/rural IRIS variation separately, when offer and demand beyond the boundary were included. Overall, for ISA midwives and gynecologists, there is more variation in rural IRIS: only 16.14 and 26.25% of rural IRIS remain unchanged for ISA midwives and gynecologists respectively, compared with 48.35 and 62.82% of urban IRIS. Moreover, a sharp downward trend was observed in the rural zone; about 53.80% of rural IRIS have a deteriorated ISA midwife and gynecologist value.

### Spatial variation of ISA according to socioeconomic distress level

The strength of the associations between the socioeconomic distress variable and composite ISA when offer and demand beyond the boundary were included or exclude are quite similar (Table 4): the association between socioeconomic factor and accessibility is therefore not impacted when offer and demand beyond the boundary Included. All the associations are positive and statistically significant (*p* < 0.0001) with the exception of the level of education; the association with women’s unemployment is close to reaching statistical significance. Population residing in the more deprived neighborhoods have the highest level of accessibility to healthcare providers, suggesting that there is no systematic absence of healthcare providers in impoverished areas.

**Table 4:** Simple linear regression between Socioeconomic variables and composite ISA when accounting or not edge effect

	ISA composite when not accounting edge effects			ISA composite when accounting edge effects		
	β	CI 95%*	*p* value	*β*	CI 95%*	*p* value
Single parent families	81.3	[72.8, 89.9]	< 0.0001	79.7	[71.3, 88.1]	< 0.0001
Non-homeowner	26.5	[23.6, 29.4]	< 0.0001	26.2	[23.4, 29.0]	< 0.0001
Insecure employment	24.9	[13.1, 36.6]	< 0.0001	27.0	[15.5, 38.5]	< 0.0001
Women’s unemployment rate	8.7	[−.3, 17.8]	0.058	9.3	[.5, 18.2]	0.04
Low level of educational attainment among women	5.0	[−3.3, 13.4]	NS 0.24	5.9	[− 2.3, 14.0]	NS 0.16

*Confidence interval at 95%

## Discussion

This work highlights the impacts of edge effect on spatial modelling of accessibility to healthcare professionals; this has been a matter of some concern to spatial analysts. Edge effect is one of the most commonly mentioned problems in studies dealing with spatial accessibility. We were interested in exploring the role of edge effect, to determine whether or not it has a relevant impact on healthcare provider accessibility in the department of Nord, using the “Index of Spatial Accessibility” previously developed by our team [44]. Our study has shown that it is difficult to reach a general conclusion. Firstly, in many published studies, authors have argued that accessibility to facilities (including healthcare providers) will lead to considerable biases [34–37], even under-reporting [17, 28, 29, 31, 43] when not accounting for the edge effect. Our work has revealed that on average, the Index of Spatial Accessibility is only slightly lower with edge effect accounted, than without. In addition, when accounting for the edge effect, our study suggests that more IRIS see their value reduced than see it improved. Indeed, when spatial analyses are not limited within a finite region, not only are facilities beyond the border disregarded, but the fact that patients from the neighboring area are also able to overcome geographical boundaries and consult a healthcare professional within the department of Nord is also ignored.

More specifically, the role of edge effect is largely linked to the method used to estimate accessibility. A range of methods exists for measurement of spatial accessibility to healthcare professionals—including Physician Population Ratio, distance/time (Euclidean, Manhattan, or network) to the nearest healthcare professional, average distance/time to a certain number of healthcare professionals, cumulative opportunity (which counts the number of opportunities that can be reached within a travel time) [22, 54] and the gravity model [23, 24]. When the accessibility indicator is based on availability or proximity (such as distance/time or cumulative opportunity) taking facilities beyond the border into account can improve the accessibility score. However, when the availability measure is weighted by population size (as our ISA indicator is), so that the volume of services available (relative to the population’s size and the proximity of services available relative to the location of the population) is taken into account, it is also important to consider demand from the population on the other side of the border. The population living either side of the study border must share the healthcare supply. As a result, the impact of edge effect on this type of accessibility indicator is more subtle; variation occurs in a balanced way, and should not be subject to arbitrary conclusions.

Secondly, our study shows that depending on health professional type, edge effect impact may vary considerably. We found that changes to GPs ISA are mainly in those IRIS located close to the boundaries. One explanation is that the “patient area” of GPs is limited (≤ 15 min) [44, 54]. Moreover, GP numbers are much higher than specialist doctor numbers, leading to more homogenous distribution. Consequently, supply and demand beyond the border will not have a very significant impact. Conversely, midwife and gynecologist numbers are very limited. People may be willing to travel further/longer to access a specialist doctor. This is why almost all IRIS are impacted by distance. Yet variations in ISA values are minor, because of the ‘balance’ of edge effects.

Healthcare accessibility is especially vital for rural populations; a matter that has long been of concern to community and health planners [17, 31–33]. Typically, these populations experience restricted access to healthcare and other resources due to the spatial inequality of living in rural or impoverished areas. ISA comparisons between urban and rural zones reveal a greater variability within the group of rural IRIS than within the group of urban IRIS. This finding may be partially explained by the spatial distribution of the rural IRIS located close to the border of the study area: 54% of rural IRIS are located within ten kilometers (as the crow flies) from the frontier (as against only 29% of urban IRIS). However, the fact that a steep downward trend was observed in the rural zone when offer and demand beyond the boundary were included is both unexpected and related specifically to the distribution of healthcare providers and consumers in the department of Nord and its neighboring areas. This result should therefore be analyzed and interpreted with caution, since it is study-area dependent. One of the explanations is that the physicians’ density of district Nord (436.2 per 100,000 inhabitants) is greater than its neighboring districts: 307.2 for Pas-de-Calais, 271.1 for Oise, 401.1 for Somme, 280.2 for Aisne and 288.5 for Ardennes [60]. On the other hand, in most cities in the Nord department, when edge effect is corrected, the ISA score is mainly classified as ‘unchanged’—thanks to well-balanced offer and demand.

We found a positive correlation between socioeconomic distress levels and composite ISA. This finding suggests that areas of high socioeconomic distress tended to have better access than low socioeconomic distress areas. This result is not surprising, given the spatial planning of the Nord department: lower-income residents are more likely to live in urban areas in which social housing and services are concentrated. This significant association is quite similar to the result when offer and demand beyond the boundary were excluded: inclusion of offer and demand beyond the boundary did not impact the relationship between distress levels and composite ISA within our study area. These findings tend to demonstrate that the impact of edge effect is dependent on both the spatial distribution of healthcare providers and territorial organization.

Our study aims to provide additional evidence to the existing scientific literature in the field of spatial accessibility to healthcare by carrying out a detailed examination of the impact of edge effect. To our knowledge, this is the first work assessing edge effect based on algorithm E2SFCA. No research has explicitly demonstrated access differences when outside healthcare sources and patient demand are excluded or included. This study highlights the fact that there is a inaccuracy in hypothesizing that accessibility will be considerably and systematically under-reported where external healthcare providers are excluded. Indeed, our study found IRIS in which the ISA was reduced when offer and demand beyond the boundary were included. The result of this study will be useful to both health resource planners and other researchers in the public health field.

Several limitations of this study should be addressed here. Despite its relative popularity of algorithm, the E2SFCA method remained highly debated. The choice of the best decay function or the right size for catchment areas needs rigorous modeling to derive the best fitting parameters [61]. In the absence of appropriate empirical evidence, it was necessary to make a number of estimations during the definition of distance-decay function and the threshold for healthcare professionals other than general practitioners.

Another limitation is aggregation error, which arises when measuring distance from aggregated areal units to facilities, and results from the use of a single point as a proxy for the locations of individuals within the area units [5]. We have attempted to reduce aggregation error by considering the spatial distribution of the living building, since it better reflects the spatial distribution of individuals [5, 62].

In this study, we were not interested in the interaction across the border between France and Belgium. Even though the European Health Insurance Card (EHIC) gives the right to access state-provided healthcare during a temporary stay in another European Economic Area, a pregnant woman have make a specific request. This request must then be accepted to be able to benefit from health care during the pregnancy and to avoid advancing their own funds to cover expenses, which do add an extra layer of administrative complexity. We assumed therefore that the offer and demand of pregnancy-related healthcare across this border is limited.

In addition, it is also worth noting that (as in many other studies dealing with spatial accessibility) our method concerns only potential spatial accessibility, rather than revealed access (actual utilization of healthcare). Only complex and expensive investigations would be capable of providing the complementary information that would allow us to distinguish the difference between spatial and real access and use of healthcare services. Finally, our study addresses difficulties arising from the use of a large amount of data and distance calculation prior to application of the algorithm, which is time consuming and calls for technical know-how. However, this is the price to be paid for a more accurate indicator.

## Conclusion

Access to healthcare services will continue to be one of the most important public health preoccupations, especially in the context of the increase of social health inequalities worldwide. Our study gave a real illustration of what could be the impact of edge effect in healthcare access in a French context. Our results did not support the “under-report” hypothesis discussed in many published studies. On the whole, our research has revealed only minor average value variations of ISA as a result of including interactions across the border. One explanation is that a kind of balance patient and healthcare professionals when considering neighboring department. However, it is not possible to set up general statement because intensity of impact varies according to healthcare provider type, urbanization level and territorial organization; in addition, we also know that the methodology implemented to measure the healthcare access combined with the size of the spatial unit may influence how the edge effect could impact the measure of healthcare accessibility. For these reason, we plan to carry out this study for another study area with a different territorial organization, to compare ISA variation in two cases in order to get a conclusion more general, at the France scale. Additional researches are required in different countries in order to improve our level of understanding about the influence of the edge effect on the accessibility to healthcare. Following the same methodology to measure the accessibility to healthcare, these different studies will help to distinguish what findings are specific to the characteristics and organization of the country and what findings are common to the different counties. It constitute a promising direction to determine more precisely healthcare shortage areas and then to fight against social health inequalities.

In conclusion, edge effect must be considered on a case-by-case basis, because it relies on choice of indicator, spatial distribution of facilities and urban organization of the territory studied.

This study represents an important step. It will serve not only to assist current researchers by identifying the common methodological hypothesis bias of edge effect in spatial accessibility studies, but will also be helpful to planners and other researchers in the public health field. This paper has presented high-quality geographic data and advanced GIS techniques. In order to examine whether the results are generalizable to different spatial scales and distribution, we hope to contribute to other areas of study in the near future.

## Figures and Tables

**Fig. 8:**
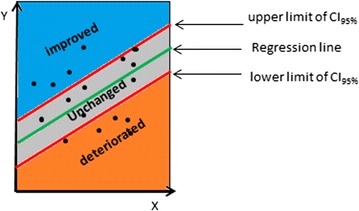
X represents ISA when not accounting for edge effect; Y represents ISA when accounting for the edge effect. Class of “unchanged” in grey color: the change between X and Y is in the average; it is points around the green regression line within the 95% confidence interval (red lines); Class deteriorated in orange color: Points placed below the lower limit of the 95% confidence interval which signifies that these IRIS have a larger reduction than the average (the reverse for “improved” in blue color)

## Appendix I

**Table:**

Socioeconomic variables	Definition
Low level of educational attainment	Proportion of women aged 25 and over not having graduated from high school
Women’s unemployment rate	Proportion of unemployed women eligible to work
Single parent families	Proportion of all households with children headed by lone parents
Non-homeowner	Proportion of all households not owning their main residence
Insecure employment situation	Proportion of those on short-term or temporary contracts, in state-funded posts, or apprenticeship/internship

## Appendix II

See Fig. 8.

## Abbreviations

E2FCA: enhanced two-step floating catchment area
INSEE: National Institute of Statistics and Economic Studies
ISA: spatial accessibility index
IRIS: L’Îlot Regroupé pour des Indicateurs Statistiques
